# Tryptophan-Containing Dual Neuroprotective Peptides: Prolyl Endopeptidase Inhibition and *Caenorhabditis elegans* Protection from β-Amyloid Peptide Toxicity

**DOI:** 10.3390/ijms19051491

**Published:** 2018-05-16

**Authors:** Paloma Manzanares, Roberto Martínez, Sandra Garrigues, Salvador Genovés, Daniel Ramón, Jose F. Marcos, Patricia Martorell

**Affiliations:** 1 Department of Biotechnology, Instituto de Agroquímica y Tecnología de Alimentos (IATA), Consejo Superior de Investigaciones Científicas (CSIC), 46980 Paterna, Valencia, Spain; sgarrigues@iata.csic.es (S.Ga.); jmarcos@iata.csic.es (J.F.M.); 2 Department of Food Biotechnology; Biópolis S.L.-Archer Daniels Midland, Parc Científic Universitat de València Edif. 2, 46980 Paterna, Valencia, Spain; roberto.martinez@adm.com (R.M.); salvador.genoves@adm.com (S.Ge.); daniel.ramonvidal@adm.com (D.R.)

**Keywords:** neurodegenerative diseases, amyloid β peptide, *Caenorhabditis elegans*, prolyl endopeptidase inhibition, lactoferrin-derived peptides, rationally-designed peptides, tryptophan, molecular docking

## Abstract

Neuroprotective peptides represent an attractive pharmacological strategy for the prevention or treatment of age-related diseases, for which there are currently few effective therapies. Lactoferrin (LF)-derived peptides (PKHs) and a set of six rationally-designed tryptophan (W)-containing heptapeptides (PACEIs) were characterized as prolyl endopeptidase (PEP) inhibitors, and their effect on β-amyloid peptide (Aβ) toxicity in a *Caenorhabditis elegans* model of Alzheimer’s disease (AD) was evaluated. Two LF-derived sequences, PKH8 and PKH11, sharing a W at the C-terminal end, and the six PACEI heptapeptides (PACEI48L to PACEI53L) exhibited significant in vitro PEP inhibition. The inhibitory peptides PKH11 and PACEI50L also alleviated Aβ-induced paralysis in the in vivo *C. elegans* model of AD. Partial or total loss of the inhibitory effect on PEP was achieved by the substitution of W residues in PKH11 and PACEI50L and correlated with the loss of protection against Aβ toxicity, pointing out the relevance of W on the neuroprotective activity. Further experiments suggest that *C. elegans* protection might not be mediated by an antioxidant mechanism but rather by inhibition of Aβ oligomerization and thus, amyloid deposition. In conclusion, novel natural and rationally-designed W-containing peptides are suitable starting leads to design effective neuroprotective agents.

## 1. Introduction

Neurodegenerative diseases account for a significant proportion of morbidity and mortality in the developed countries. Moreover, these disorders are becoming more frequent due to the increased life expectancy [1]. Alzheimer’s disease (AD), the most common type of senile dementia, is a neurodegenerative disorder with enormous social and economic impact [2]. Although the cause(s) of AD is still controversial, it is accepted that the accumulation of amyloid β peptide (Aβ) in brain plaques triggers downstream neurotoxic events, leading to neuronal dysfunction, cell death, and neurodegeneration [3]. Aβ is a 38–43 amino acid peptide which derives from the β-amyloid precursor protein (βAPP) through proteolytic processing by β- and γ-secretases. Aβ 1–42 (Aβ_1–42_) appears to be the species that first forms deposits in AD [2], and its role in the oxidative damage in AD brain has also been established [4]. These findings suggest amyloid plaques or Aβ production as targets for drug development [1]. In addition, compounds that exhibit antioxidant activity can be considered potential therapeutic agents for AD [5]. In this context, the nematode *Caenorhabditis elegans* engineered to express human Aβ_1–42_ is a convenient in vivo model that has been used in drug screening for potential AD therapeutics. The resulting transgenic strains develop a concomitant progressive paralysis phenotype, being a well-suited model for correlating Aβ expression and toxicity [6,7,8].

Prolyl endopeptidase (PEP; E.C. 3.4.21.26) also called prolyl oligopeptidase (POP) is a highly conserved serine protease enzyme that cleaves peptide bonds at the carboxyl side of proline residues (P) in peptides up to 30 amino acids long. It has been found in a wide range of species and tissues, especially in the human brain. PEP can degrade biologically active P-containing peptides, including peptide-like hormones, neuroactive peptides, and various cellular factors [9]. Moreover, levels of PEP activity are altered in many degenerative conditions and psychiatric disorders, such as AD, amnesia, depression, and schizophrenia, and therefore, the enzyme is a potential therapeutic target for these diseases [10]. PEP was suggested to be a putative γ-secretase and accordingly PEP inhibitors abolished the formation of Aβ in neuroblastoma cells and prevented amyloid deposition in a mouse model of accelerated senescence [11]. However, PEP is not only involved in cleaving off physiologically active peptides, and it has been speculated that its physiological role results from its direct interaction with partner proteins [12].

The potential therapeutic use of peptides derived from natural sources has been extensively discussed during the last decades [13,14,15]. Dietary peptides have been claimed to have positive effects on weight loss and glycemia management [16] and also in the prevention and treatment of cancer, cardiovascular and infective diseases, and mental health disorders [17,18]. Moreover, many peptides seem to act through more than a single mechanism of action and, hence, they can be considered multifunctional sequences [19,20]. Natural peptides also represent an excellent starting point as leading candidates for the rational design of synthetic sequences with improved biological activity, specificity, and stability [21].

Natural sources of PEP-inhibitory peptides mainly include meat and fish by-products [22,23,24,25,26], cereals [27], and milk proteins [25,28,29,30]. By contrast, peptides showing protection against the toxicity caused by the accumulation of Aβ are scarce. These potential neuroprotective peptides were isolated from a cocoa by-product [31], maize [32], and scorpion venom [33]. Furthermore, small rationally-designed peptides based on specific Aβ motifs able to interact with Aβ, modifying its kinetics of aggregation, and reducing its toxicity have been described as an attractive pharmacological strategy [34,35].

Lactoferrin (LF), a well-characterized component of milk whey, is a multifunctional iron glycoprotein that exhibits a diverse range of biological activities including antimicrobial, antiviral, antioxidant, and immunomodulatory activities [36]. LF-derived peptides share some biological activities with the intact protein and possess antihypertensive properties [20]. Recently, we have shown the inhibitory effect of an LF-based product on Aβ toxicity [37], but there is no information about the neuroprotective effects of LF-derived peptides.

In the present study, we investigated the PEP-inhibitory activity of LF-derived peptides and of a set of sequence-related synthetic heptapeptides. Peptides exhibiting PEP-inhibitory activity in vitro were examined in a transgenic *C. elegans* model of AD to evaluate their in vivo protective effects against Aβ toxicity. With the aim of characterizing the mechanism(s) mediating *C. elegans* protection, we further examined their in vivo antioxidant effect and their in silico molecular interactions with Aβ. Finally, the role of tryptophan residues (W) on the neuroprotective activity of peptides was investigated.

## 2. Results

### 2.1. Lactoferrin Hydrolysates Inhibit Prolyl Endopeptidase Activity

**Figure 1:**
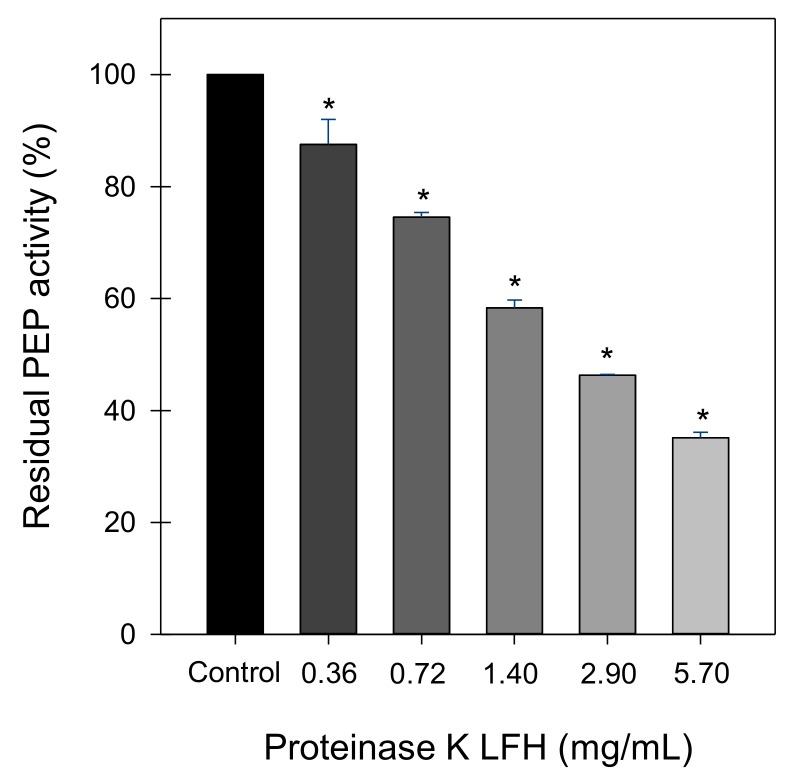
The concentration-dependent effect of lactoferrin hydrolysate (LFH) generated by proteinase K on prolyl endopeptidase (PEP) residual activity. Data are expressed as the mean ± SD of three replicates. * Significantly different from the control (*p* < 0.01; one-way ANOVA followed by a Dunnett multiple comparison test).

LF hydrolysates (LFH) generated by pepsin, proteinase K, or trypsin and subjected to ultrafiltration through a 3 kDa cut-off membrane showed PEP inhibitory activity (Table 1). At the maximum concentration tested (2 mg/mL), proteinase K LFH provoked the highest inhibitory effect (41% of inhibition) while pepsin and trypsin hydrolysates inhibited PEP by approximately 20%. The proteinase K LFH (0.36–5.7 mg/mL) provoked significant concentration-dependent inhibition of PEP (Figure 1), reaching 65% inhibition at the highest concentration assayed (5.7 mg/mL). Non-hydrolysed LF (0.7 and 1.4 mg/mL) did not show any inhibitory effect on PEP activity.

### 2.2. Lactoferrin-Derived Peptides from Proteinase K Hydrolysate Are Prolyl Endopeptidase Inhibitors

**Figure 2:**
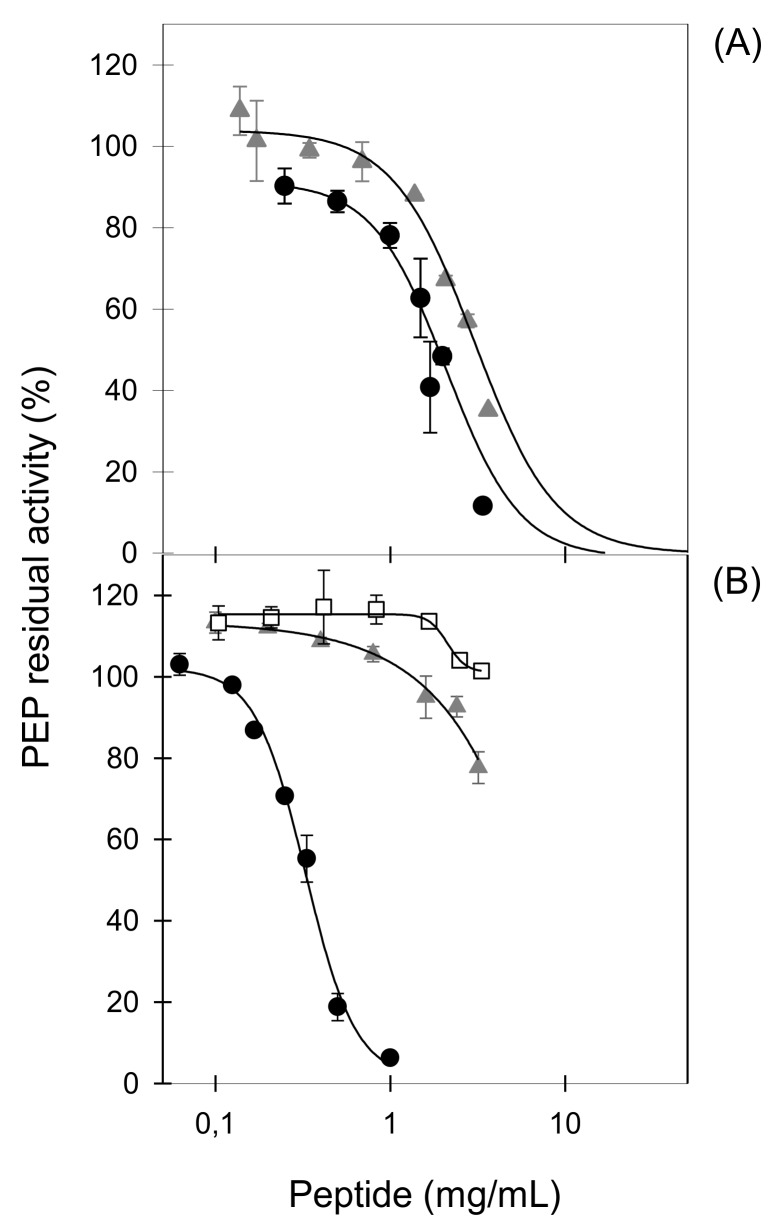
The concentration-dependent effect on prolyl endopeptidase (PEP) residual activity of peptides PKH11 and PACEI50L and their sequence variants obtained by W replacement with A. (**A**) PKH11 (SVDGKEDLIW; black circles) and its sequence variant PKH11-v1 (SVDGKEDLIA; grey triangles); (**B**) PACEI50L (RKWHFLW; black circles), and its two sequence variants PACEI50L-v1 (RKWHFLA; grey triangles) and PACEI50L-v2 (RKAHFLA; white squares).

Proteinase K LFH is a complex hydrolysate from which 37 peptides were identified by high performance liquid chromatography tandem mass spectrometry (HPLC MS/MS) [38]. Eleven of the most abundant peptides identified in the hydrolysate were chemically synthesized and their in vitro PEP-inhibitory activity was tested (Table 2). Six out of eleven peptides (PKH3, PKH4, PKH5, PKH6, PKH9, and PKH10) included one P residue in the sequence but as summarized in Table 2, all of them failed to inhibit PEP. Only two sequences, the hexapeptide PKH8 (NEGLTW) and the decapeptide PKH11 (SVDGKEDLIW), which share a W residue at the C-terminal end, exhibited significant inhibitory activities of 12% and 22%, respectively, in the conditions tested. Further experiments were carried out to determine the inhibitory potency of the most promising PEP-inhibitory peptide, PKH11, which showed an IC_50_ value of 2.1 ± 0.2 mg/mL (Figure 2A).

### 2.3. Tryptophan-Containing Synthetic Heptapeptides Inhibit Prolyl Endopeptidase

The potential role of a W residue at the C-terminal end of LF-derived PEP-inhibitory peptides has also been pointed out in angiotensin-converting enzyme (ACE) inhibitory peptides, in which W at the C-terminus is associated with high inhibitory potency [20]. This prompted us to evaluate a set of six synthetic heptapeptides, which share two W residues at positions 3 and 7 (Table 3) and previously described as ACE inhibitors [39]. The inhibitory effects of 1 mg/mL heptapeptides on PEP activity are summarized in Table 3. The six heptapeptides showed significant enzyme inhibition with values ranging from 29% to 94%. Remarkably, PACEI50L exhibited a potent PEP-inhibitory activity with an IC_50_ value as low as 0.33 ± 0.02 mg/mL (Figure 2B).

### 2.4. Tryptophan Residues Are Important for Prolyl Endopeptidase Inhibition in PACEI50L and PKH11

To characterize the role of the C-terminal W in PEP-inhibitory activity, this amino acid residue was exchanged to alanine (A) in the selected sequences PACEI50L and PKH11 and thus the sequence variants RKWHFLA (PACEI50L-v1) and SVDGKEDLIA (PKH11-v1) were generated. A second PACEI50L derivative (PACEI50L-v2; RKAHFLA) was obtained by replacing both W residues at position 3 and 7 with A. We next analyzed the impact of the amino acid exchanges on PEP inhibition (Figure 2). The exchange of W to A moderately reduced PEP inhibition by PKH11-v1, in which the inhibitory potency (IC_50_ = 3.0 ± 0.3 mg/mL) was significantly lower than the one showed by the natural peptide PKH11 (IC_50_ = 2.1 ± 0.2 mg/mL), as can be seen in Figure 2A. By contrast, a severe reduction of the inhibitory activity of both PACEI50L variants was observed (Figure 2B). At the highest concentration tested (3.3 mg/mL), PACEI50L-v1 inhibited PEP by 20% while PACEI50L-v2 did not exhibit a clear inhibitory effect (Figure 2B).

### 2.5. Prolyl Endopeptidase-Inhibitory Peptides Alleviate Amyloid β Peptide-Induced Paralysis in the Transgenic C. elegans

To study the functional effect of PEP-inhibitory peptides in an in vivo AD model, we used the transgenic *C. elegans* strain CL4176 engineered to express human Aβ_1–42_. The active peptides PACEI50L and PKH11 were assayed at doses of 0.5, 0.1, and 0.02 µg/mL. Statistical analysis showed significant differences among paralysis curves of control and treated worms at doses of 0.5 and 0.1 µg/mL, thus, indicating a delay in paralysis (Table 4). The addition of PACEI50L and PKH11 at 0.5 and 0.1 µg/mL to the *C. elegans* medium provoked a reduction in the percentage of paralyzed worms at the end of the assay (49 h) and, in some cases, also a delay in the onset paralysis (Table 4). The dose of 0.02 µg/mL did not provide any effect (data not shown). Moreover, the most effective dose was 0.1 µg/mL for both PACEI50L and PKH11 peptides (Trial 2, PACEI50L 0.1 versus PACEI50L 0.5, *p* = 0.004; Trial 2, PKH11 0.1 versus pKH11 0.5, *p* = 0.006; paired log Rank survival test).

**Figure 3:**
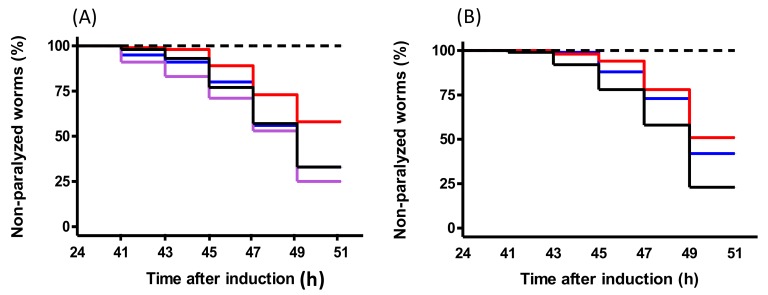
The body paralysis of *C. elegans* CL4176 measured after the temperature up-shift in nematodes treated with or without peptides; worms without temperature induction were included as the negative control (dashed line). Time refers to the hours after Aβ_1–42_ expression induced by the temperature up-shift. (**A**) Non-treated worms (black line); treated-worms with 0.1 µg/mL of PACEI50L (red line), 0.1 µg/mL PACEI50L-v1 (blue line), and 0.1 µg/mL PACEI50L-v2 (purple line); data are the average of three independent trials. (**B**) Non-treated worms (black line); treated-worms with 0.1 µg/ml of PKH11 (red line) and 0.1 µg/mL PKH11-v1.

The average paralysis curves of 0.1 µg/mL PACEI50L and PKH11 treatments are shown in Figure 3, and the statistical analyses of the curves are summarized in Table 5 and Table 6. In the case of peptide PACEI50L, a decrease of 25.2% of paralyzed worms compared with control fed nematodes at 49 h was observed (Figure 3A and Table 5) whereas a delay in the onset paralysis and a reduction of 20% of final paralyzed worms were noticed for PKH11 treatment (Figure 3B and Table 6).

We also evaluated the activity of both peptide variants derived from PACEI50L at the effective dose of 0.1 µg/mL. Both modified peptides significantly changed their activity when compared with the original PACEI50L peptide, and no protective effect upon paralysis was detected (Table 4 and Table 5, and Figure 3A). In the case of PACEI50L-v2, the final percentage of non-paralyzed worms was even 5.6% lower than the control conditions. Finally, the analysis of the modified peptide PKH11-v1 (0.1 µg/mL) resulted in a partial reduction of the efficacy upon paralysis (Table 4 and Table 6, and Figure 3B), in correlation with the in vitro data of PEP inhibition.

### 2.6. Peptides Do Not Protect C. elegans from Oxidative Stress

**Figure 4:**
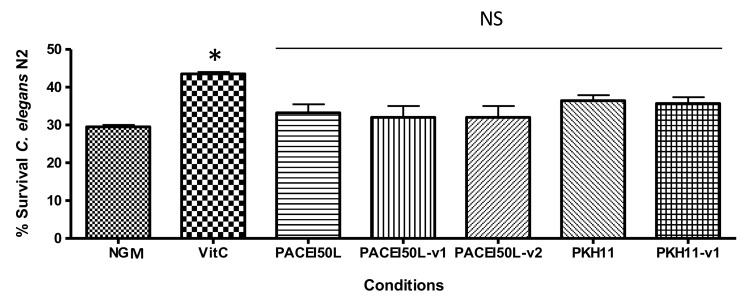
The percentage of *C. elegans* N2 survival after an acute oxidative stress with H_2_O_2_. Nematodes were cultured in control conditions (NGM), with vitamin C (10 µg/mL) and with peptides (0.1 µg/mL). * Significantly different from the control (*p* < 0.05; one-way ANOVA followed by Tukey’s HSD); NS: not significant (*p* > 0.05).

The protective effect upon oxidative stress in the wild-type strain N2 of *C. elegans* was assayed with PACEI50L, PKH11, and the corresponding peptide variants at a concentration of 0.1 µg/mL. Figure 4 shows that none of them was able to increase the worm survival after induction of oxidative stress with 2 mM H_2_O_2_, in contrast to the significant protection caused by vitamin C. These results indicate that peptides tested at 0.1 µg/mL, which is the most effective dose reducing *C. elegans* paralysis, did not show any antioxidant effect.

### 2.7. Molecular Docking Analyses Reveal Different Intermolecular Interactions with Aβ_1–42_ Depending on the Peptide Sequence

**Figure 5:**
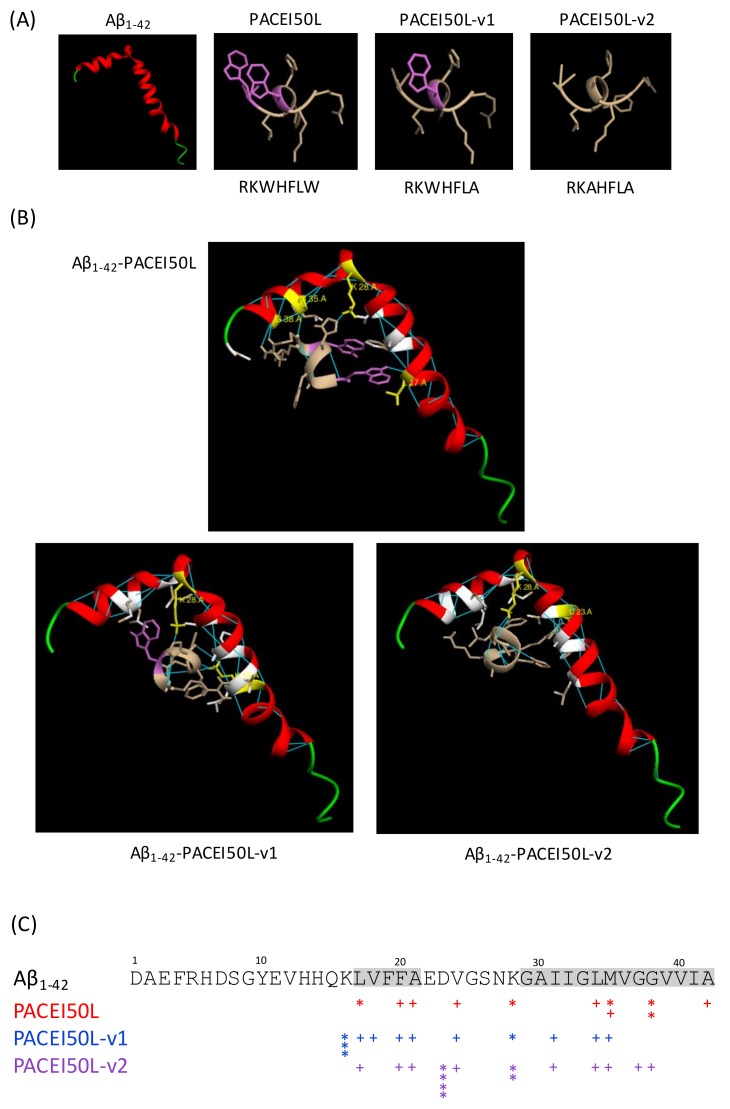
The molecular docking model of the Aβ_1–42_ monomer binding to PACEI50L, PACEI50L-v1, and PACEI50L-v2. (**A**) The structure of Aβ_1–42_ (PDB, ID:1IYT), PACEI50L, PACEI50L-v1, and PACEI50L-v2; W residues are in purple. (**B**) PACEI50L is bound to Aβ_1–42_ via five intermolecular H-bonds with L17, K28, M35, and G38. PACEI50L-v1 is bound to Aβ_1–42_ via four H-bonds with K16 and K28. PACEI50L-v2 is bound via six intermolecular H-bonds with D23 and K28; Aβ_1–42_ residues participating in molecular interactions are in yellow (H bonds) and white (hydrophobic interactions); H-bonds are in blue. (**C**) A schematic representation of molecular interactions between Aβ_1–42_ and PACEI50L (red), PACEI50L-v1 (blue), and PACEI50L-v2 (purple); * intermolecular H-bond, + hydrophobic interaction, hydrophobic domains in Aβ_1–42_ are highlighted in grey.

To examine the interaction between the peptides and Aβ_1–42_, molecular docking simulations were performed. Since the W replacement with A in the PACEI50L sequence abolished the protective effect in *C. elegans*, PACEI50L and its two inactive variants, PACEI50L-v1 and PACEI50L-v2, were chosen to gain insight into how peptides might interact with Aβ_1–42_. Peptide and Aβ_1–42_ monomer (ID: 1IYT) structures are shown in Figure 5A, and the simulated interactions obtained from the docking analyses are shown in Figure 5B. Figure 5C summarizes the molecular interactions in the three complexes. In the most probable model, the complex Aβ_1–42_-PACEI50L is stabilized by five intermolecular H-bonds: two H-bonds between R1 of PACEI50L and G38 of Aβ_1–42_ (R1-G38), one H-bond R1-M35, one H-bond H4-K28, and one H-bond W7-L17. PACEI50L also interacts through hydrophobic interactions with other residues of Aβ_1–42_ (F20/A21/V24/L34/M35/G38/A42). The interaction between Aβ_1–42_ and the two inactive variants is qualitatively different. The interaction with PACEI50L-v1 is stabilized by H-bonds involving K16 and K28 residues from Aβ_1–42_: one H-bond H4-K28, one H-bond L6-K16, and two H-bonds A7-K16. In this complex, hydrophobic interactions include residues L17/V18/F20/A21/V24/I31/L34/M35. PACEI50L-v2 docked to Aβ_1–42_ revealed 6 H-bonds via D23 and K28: four H-bonds R1-D23 and two H-bonds L6-K28. The binding is also stabilized by hydrophobic interactions through L17/F20/A21/V24/I31/L34/M35/G37/G38. Moreover, both inactive variants of PACEI50L change their positioning relative to Aβ_1–42_ when compared to the parental peptide.

## 3. Discussion

Currently, peptides have a wide range of applications in medicine and biotechnology. Moreover, multifunctionality is a common trait of many peptides, which might function as polypharmacological sequences. The present study characterizes new dual neuroprotective peptides that show in vitro PEP-inhibitory properties and *C. elegans* protection against Aβ_1–42_-associated toxicity in vivo. The neuroprotective peptides include natural sequences derived from the milk protein LF and sequence-related rationally-designed synthetic heptapeptides.

LFHs obtained with three different proteases showed a moderate ability to inhibit PEP that was not shown by non-hydrolysed LF, suggesting that LF-derived peptides possess the inhibitory activity. The hydrolysates generated with three different proteases showed different PEP-inhibitory activities pointing out the importance of peptide sequences for enzyme inhibiting activity. In our study, the most potent LFH was generated using proteinase K, which exhibits specificity for peptide bonds adjacent to the carboxylic group of aliphatic and aromatic amino acids [40]. Moreover, the inhibitory activity of the hydrolysate was comparable to the previously reported IC_50_ values of a sodium caseinate hydrolysate (0.77 mg/mL) [29] and different fish protein hydrolysates (1.10–4.21 mg/mL) [23]. LFHs obtained with proteinase K were previously described as in vitro inhibitors of the endothelin-converting enzyme (ECE) and ex vivo as inhibitors of ECE-dependent vasoconstriction [38]. Our results describe a new biological activity related to neuroprotection for LF-derived peptides.

PEP inhibitors developed as potential therapeutic drugs are substrate-like compounds containing one P or one P-analogue residue [10]. Most of the PEP-inhibitory peptides derived from natural sources described to date contain at least one and up to six P residues in their sequences, and from 3 to 18 amino acid residues in length [22,27,29]. In spite of the fact that six of the eleven LF-derived peptides evaluated here contain one P residue in their sequences, none of them displayed PEP-inhibitory activity, as shown for some P-containing peptides derived from collagen or corn γ-zein [27]. By contrast, the only two LF-derived peptides that provoked a modest in vitro enzyme inhibition (PKH8, NEGLTW; PKH11, SVDGKEDLIW) do not contain any P residue in their sequence, suggesting that both LF-derived peptides do not act as substrate-type inhibitors. Non-P-containing peptides derived from α-casein were also described as PEP inhibitors [30].

Since the two LF-derived peptides with PEP-inhibitory activity described here shared a C-terminal W, we hypothesized its potential key contribution to enzyme inhibition. Our hypothesis was confirmed by the ability of the six sequence-related synthetic heptapeptides (PACEI48L to PACEI53L) to inhibit PEP. PACEI heptapeptides are the second generation of angiotensin converting enzyme inhibitory peptides based on two hexapeptides leads, PACEI32L (RKWHFW) and PACEI34L (RKWLFW) [41]. Heptapeptides were designed by combinations of F, H, and L residues in positions 4–6, all of them share R, K, and W residues at the N-terminus and W residue at the C-terminus of a given heptapeptide (Table 3) [39]. Among these peptides, the highest inhibitory activity was recorded for PACEI50L with H, F, and L residues at positions 4, 5, and 6, respectively. The activity of this heptapeptide is sequence-specific, as demonstrated by the swapping of H and F residues (sequences PACEI50L and PACEI48L) which drastically reduced the PEP-inhibitory activity. These results confirm and extend previously reported data on how minor amino acid exchanges affect biological properties [41,42].

Further confirmation of the key role of W residue at the C-terminus was provided by the evaluation of PACEI50L and PKH11 variants that contain W to A substitutions. Our results showed that the effect of W residue on PEP-inhibitory activity is dependent on the peptide sequence since a severe reduction of the biological activity was observed for the two variants of PACEI50L while only a 40% reduction of the inhibitory potency was recorded for the PKH11 variant. Our results also pointed to the relevance of both W residues in the PACEI50L sequence since the total loss of PEP inhibition was achieved only with the double substitution of W residues at positions 3 and 7. In agreement with these results, the most potent PEP-inhibitory peptides named 13L and 9L identified from a cocoa hydrolysate contained W in their sequence (13L, DNYDNSAGKWWVT; 9L, NYDNSAGKW) and the lower IC_50_ value (0.19 mg/mL) corresponded to the sequence 13L [31]. Additional studies are required to explain the role of W residues in PEP-inhibitory peptides.

*C. elegans* is a suitable in vivo model for research on the molecular biology and genetics of different diseases as well as for drug and bioactive compound screenings [43]. *C. elegans* models present alternative approaches to understanding neurodegenerative diseases for which there are currently few effective therapies [6]. Here the transgenic *C. elegans* model of AD, which develops a paralysis phenotype, was used to study the effects of peptides. PACEI50L and PKH11 treatments ameliorated Aβ-induced paralysis suggesting a potential in vivo protection from Aβ_1–42_ toxicity, as described for cocoa and maize peptides [31,32] and also for a sequence purified from scorpion venom [33]. Remarkably, and as observed for PEP-inhibitory activity, peptide variants of PACEI50L completely lost paralysis suppression in *C. elegans* demonstrating the positive role of W residues in the context of the heptapeptide sequence evaluated.

The effect of different compounds on Aβ_1–42_–mediated paralysis in transgenic *C. elegans* has also been associated with antioxidant effects [31,32,33,44]. In contrast to other neuroprotective peptides, the reduction of Aβ-toxicity by PACEI50L and PKH11 does not seem to operate through an antioxidant mechanism since both peptides did not produce significant *C. elegans* protection upon oxidative stress under the conditions tested. Conversely, the antioxidant properties of maize and scorpion venom peptides might underline, at least in part, their protective effect observed in *C. elegans*, since they inhibited the production of reactive oxygen species [32,33]. Additionally, in a similar experiment to the one described here, the peptide 13L from cocoa provided protection against oxidative stress, suggesting that the antioxidant activity might contribute to the protection against Aβ_1–42_-induced damage [31]. Therefore, our results suggest alternative mechanisms for PACEI50L and PKH11.

Aβ_1–42_ contains hydrophobic motifs in the central (residues 17–21) and carboxy-terminal (residues 29–42) regions of the peptide [45]. Amyloidogenic peptide aggregation seems to be primarily driven by these hydrophobic domains [46]. Here, docking analysis showed that the potential hydrogen bond interactions between the active PACEI50L and Aβ_1–42_ are focused on amino acids within both hydrophobic motifs (L17, M35, and G38) or close to them (K28). Particularly M35 and its hydrophobic surroundings seem to be important for the oxidative, neurotoxic, and aggregation properties of the peptide [47,48] whereas G residues can stabilize amyloidogenic structures by means of hydrogen bonds [49]. The conformation adopted by Aβ_1–42_ seems to be an important factor in amyloid formation since the Aβ_1–42_ peptide with α-helical or random coil structure aggregates slowly while Aβ with β-sheet conformation aggregates rapidly [45]. L17 included in the central hydrophobic stretch 17–21 contributes to adopt a β-sheet conformation that facilitates monomeric interaction, β-sheet oligomers, and amyloid fibrils [50]. Moreover, amino acids 17–20 served as a template for designing synthetic peptides able to inhibit fibrillogenesis in a rat brain model of amyloidosis [35]. Our docking results suggest that molecular interactions through hydrogen bonding between PACEI50L and residues L17, M35, and G38 from Aβ_1–42_ might impede monomeric interactions and thus, Aβ oligomerization. Docking analysis revealed the loss of hydrogen bonds between non-active PACEI50L variants and these three residues in Aβ_1–42_. Whether PACEI50L might interfere with the folding of Aβ_1–42_ to form aggregates as suggested by in silico analysis requires further in vitro and in vivo research.

Our study underscores the important role of W for the bioactivity of neuroprotective peptides. W-containing peptides as those described here display several biological activities including antihypertensive, antioxidant, antidiabetic, and satiating properties [51]. Besides W is the sole precursor of serotonin, which has been reported to have an effect on the psychological/cognitive function in humans. Remarkably, increased dietary W intake reduced intra-neuronal Aβ accumulation in a mouse model of AD, suggesting a neuroprotective role of W through the increase of serotonin levels [52]. Increased W levels also extend longevity in *C. elegans* and protect from alpha-synuclein and polyglutamine toxicity [53,54], suggesting a protective role against proteotoxicity in aging and age-related diseases. Additionally, in the *C. elegans* model, supplementation with the W-containing 13L peptide upregulated the W metabolism, including genes involved in the synthesis of serotonin and other neurotransmitters [31]. It is worthwhile to note that milk proteins and, among them, LF, are particularly rich in W in comparison with other dietary proteins opening the way to the future inclusion of LF in dietary recommendations for preventing or postponing AD.

In conclusion, we have identified novel natural and rationally-designed W-containing peptides showing in vitro PEP inhibition and in vivo protection from Aβ_1–42_ toxicity, although further research needs to be conducted in murine models to analyze the effectiveness of the peptides. These results add a new application to the antihypertensive proteinase K LFH and the synthetic heptapeptides confirming their multifunctionality. The effect of PACEI50L on delayed paralysis in *C. elegans* might be mediated, at least in part by the inhibition of Aβ_1–42_ oligomerization and thus, amyloid deposition, while the peptide antioxidant activity does not seem to be involved in the protective effect. Our results suggest that W-containing peptides are suitable starting leads to design effective neuroprotective agents. Further improvements to increase in vivo peptide stability based on the use of D-amino acid sequences are in progress. Future efforts are currently directed to clarify the mechanisms underlying the in vivo protective effects of W-containing peptides.

## 4. Materials and Methods

### 4.1. Materials

Bovine LF was provided by FrieslandCampina Domo (Zwolle, The Netherlands). Porcine pepsin and trypsin (type II-S), the bicinchoninic acid (BCA) kit, and the PEP substrate Z-Gly-Pro-*p*-nitroanilide were purchased from Sigma (Madrid, Spain). PEP was supplied by the Seikagaku Corporation (Tokyo, Japan). Recombinant proteinase K was purchased from Roche (Mannheim, Germany).

### 4.2. Lactoferrin Hydrolysates and Peptides

Bovine LF (5% *w*/*v*) was hydrolyzed using pepsin (3% *w*/*w*), trypsin (1% *w*/*w*), or proteinase K (1% *w*/*v*) as previously described [38,55]. LFHs were subjected to ultrafiltration through a polyethersulfone membrane with a 3 kDa cut-off (Vivascience, Sartorius Stedim Biotech, Aubagne, France) and the permeates were kept at −20 °C until use.

Synthetic peptides were purchased at >95% purity from GenScript Corporation (Piscataway, NJ, USA), wherein they were synthesized by solid phase methods using *N*-(9-fluorenyl) methoxycarbonyl chemistry. PACEI peptides were acetylated at the N-terminus and amidated at the C-terminus. The synthetic peptide concentration was based on the dry weight and purity provided by the manufacturer.

The protein content of LFHs was estimated by the BCA method using bovine serum albumin as the standard [55].

### 4.3. Prolyl Endopeptidase Assay

The PEP activity was determined as previously described [31] with minor modifications. Enzyme assays were performed in 96-well microplates using Z-Gly-Pro-*p*-nitroanilide as a substrate and measuring the increase in absorbance at 410 nm due to the release of *p*-nitroaniline. Ten µL of tenfold peptide or hydrolysate solution in 100 mM sodium phosphate buffer, pH 7 (final concentrations from 0.063 to 3.6 mg/mL for peptides and from 0.35 to 5.7 mg/L for LFHs), 30 µL of a 0.1 U/mL PEP solution in 100 mM sodium phosphate buffer (pH 7) and 35 µL of the same sodium phosphate buffer were pre-incubated at 30 °C for 5 min, and the mixture incubated with 25 µL of 2 mM Z-Gly-Pro-*p*-nitroanilide in 40% 1,4-dioxane for 30 min at the same temperature. The reaction was finished by the addition of 100 µL 10% Triton X-100 in 1 M sodium acetate buffer (pH 4), and the absorbance measured at 410 nm in a microplate reader. Data are expressed as the percentage of PEP residual activity with respect to a control without peptide (100%).

The IC_50_ value of a peptide was defined as the concentration required to inhibit 50% of the PEP activity and the value for each experiment was estimated by non-linear regression of the experimental data to a four-parameter logistic curve using the software package SigmaPlot v 13.0 (SPSS Inc., Chicago, IL, USA).

### 4.4. C. elegans Strains and Maintenance

Wild-type Bristol strain N2 and the transgenic strain CL4176 (smg-1^ts^ [pAF29(*myo-3*/Aβ_1–42_/let UTR) + pRF4(*rol-6*(*su10069*))]) were obtained from the *Caenorhabditis* Genetics Center (College of Biological Sciences, University of Minnesota, Saint Paul, MN, USA) and were used for oxidative stress experiments and paralysis assays, respectively. *C. elegans* strains were routinely propagated on Nematode Growth Medium (NGM) plates with *Escherichia coli* strain OP50 as a food source at 20 °C (N2) or at 16 °C (CL4176). Worms were synchronized by isolating eggs from gravid adults at 20 °C (N2) or at 16 °C (CL4176), and eggs were hatched overnight in NGM plates.

In the oxidative and paralysis experiments, worms were fed with the different compounds from egg to adult stages, and transferred to new plates every two days.

### 4.5. C. elegans Paralysis Assay

Paralysis is induced in strain CL4176 by the expression of a muscle-specific Aβ_1–42_ which depends on up-shifting temperature from 16 to 25 °C [7,8]. For paralysis experiments, strain CL4176 maintained at 16 °C was egg-synchronized in plates containing NGM (control medium) and NGM with peptides (0.02–5 µg/mL). Transgene expression was induced by up-shifting the temperature from 16 to 25 °C, starting 24 h after egg laying and maintained for 24 h. The worms were incubated at 20 °C until all the worms in the experiment became paralyzed. Paralysis was scored 24 h after induction. Paralysis in induced worms was compared with non-induced worms (maintained at 16 °C until the end of the paralysis assay). Experiments were carried out at least in duplicate.

### 4.6. C. elegans Oxidative Stress Assays

For oxidative stress experiments, strain N2 was egg-synchronized in NGM plates (control medium) and NGM supplemented with 0.1 µg/mL peptides or 0.1 mg/mL vitamin C (positive control). The viability of *C. elegans* was assessed after 2 mM hydrogen peroxide (H_2_O_2_)-induced oxidative stress for 5 h [56]. Experiments were carried out in triplicate.

### 4.7. Molecular Docking

Structural modeling of the linear peptide PACEI50L and its variants PACEI50L-v1 and PACEI50L-v2 was carried out using the PEP-FOLD3 platform (Université Paris Diderot, Paris, France) [57] currently available on the RPBS web portal (http://mobyle.rpbs.univ-paris-diderot.fr). Solution structure of Aβ_1–42_ was obtained from the Protein Data Bank (PDB, ID: 1IYT). The molecular docking of the peptides to Aβ_1–42_ was performed using the ClusPro web server (Boston University, Boston, MA, USA; https://cluspro.bu.edu) [58]. ClusPro model selection is based on the cluster size rather than on the cluster energy score. Model 0 was selected for complexes Aβ_1–42_-PACEI50L (cluster size, 834; cluster score, −573.1), Aβ_1–42_-PACEI50L-v1 (cluster size, 642; cluster score, −549.7), and Aβ_1–42_-PACEI50L-v2 (cluster size, 218; cluster score, −457.4). The 3D models of the peptides and their interactions were visualized by the UCSF Chimera software (University of California, San Francisco, CA, USA) [59].

### 4.8. Statistics

Statistical analyses were carried out using the GraphPad Prism 4 software package (GraphPad Software, La Jolla, CA, USA). PEP residual activity data are mean ± standard deviation (SD) and were subjected to either Student’s *t*-test or one-way ANOVA followed by Dunnett post-test or Tukey’s honestly significant difference procedure (HSD). The comparison of the *C. elegans* paralysis curves was performed using the log-rank survival test. The survival data of the *C. elegans* was assessed after oxidative stress and differences between nematodes cultured in the control and treatment conditions were evaluated by means of a one-way ANOVA followed by Tukey’s HSD. *p* < 0.05 was considered significant.

## Figures and Tables

**Table 1:** Effects of lactoferrin hydrolysates (LFH) on prolyl endopeptidase (PEP) activity.

LFH	Concentration (mg/mL)	PEP Residual Activity (%) ^1^
Pepsin LFH	1	94.7 ± 0.1
	2	84.1 ± 2.9 **
Proteinase K LFH	1	76.6 ± 0.3 **
	2	59.4 ± 1.2 **
Trypsin LFH	1	90.0 ± 0.3 *
	2	81.5 ± 0.6 **

^1^ Data are expressed as the percentage of PEP residual activity with respect to a control without peptide (100%) and are the mean ± standard deviation (SD) of three replicates. * Significant inhibition with respect to control (*p* < 0.05); ** Significant inhibition with respect to control (*p* < 0.01) (Student’s *t*-test on absolute values of PEP activity).

**Table 2:** Effects of peptides isolated from proteinase K lactoferrin hydrolysate (LFH) on prolyl endopeptidase (PEP) activity.

Peptide (1 mg/mL)	Sequence	PEP Residual Activity (%) ^1^
PKH1	DRDQY	102.7 ± 7.0
PKH2	VVKKGSNF	115.0 ± 1.0 **
PKH3	ENLPEKA	97.1 ± 0.1
PKH4	RIPSKV	109.6 ± 2.2 *
PKH5	GGRPTYEEY	96.9 ± 2.1
PKH6	GILRPY	97.2 ± 4.0
PKH7	DRDQYELL	115.3 ± 4.2 **
PKH8	NEGLTW	87.1 ± 1.0 **
PKH9	NIPMGL	95.5 ± 3.4
PKH10	GILRPYL	103.9 ± 0.8
PKH11	SVDGKEDLIW	78.0 ± 3.1 **

^1^ Data are expressed as the percentage of PEP residual activity with respect to a control without peptide (100%) and are the mean ± SD of three replicates. * Significant inhibition with respect to control (*p* < 0.05). ** Significant inhibition with respect to control (*p* < 0.01) (Student’s *t*-test on absolute values of PEP activity).

**Table 3:** Effects of heptapeptides (PACEI) on prolyl endopeptidase (PEP) activity.

Peptide (1 mg/mL)	Sequence ^1^	PEP Residual Activity (%) ^2^
PACEI48L	Ac-RKWFHLW-NH_2_	61.4 ± 4.4 ** (a)
PACEI49L	Ac-RKWFLHW-NH_2_	46.8 ± 9.1 ** (b)
PACEI50L	Ac-RKWHFLW-NH_2_	6.4 ± 2.3 ** (c)
PACEI51L	Ac-RKWHLFW-NH_2_	71.6 ± 4.9 ** (a)
PACEI52L	Ac-RKWLFHW-NH_2_	65.9 ± 3.6 ** (a)
PACEI53L	Ac-RKWLHFW-NH_2_	71.5 ± 4.5 ** (a)

^1^ All the peptides were acetylated at the N-terminus (Ac) and amidated at the C-terminus (NH_2_). ^2^ Data are expressed as the percentage of PEP residual activity with respect to a control without peptide (100%) and are the mean ± SD of three replicates. ** Significant inhibition with respect to control (*p* < 0.01) (Student’s *t*-test on absolute values of PEP activity). Data with the same letter do not differ at the 95% level of confidence (Tukey’s honestly significant difference (HSD) procedure).

**Table 4:** Effects of peptide treatments on the CL4176 paralysis and statistical analyses of the paralysis curves ^a^.

Treatment	Dose	Trial	Strain	Nematodes (n)	Onset Paralysis ^b^	Paralyzed Worms ^c^	Log Rank Χ^2^	*p*-Value
PACEI50L	0.5	1	NGM	110	41 (98.2%)	70.0%		
			NGM + PACEI50L	92	45 (96.7%)	41.3%	16.65	<0.0001
		2	NGM	113	43 (94.7%)	68.1%		
			NGM + PACEI50L	120	43 (99.2%)	41.7%	16.94	<0.0001
	0.1	1	NGM	181	43 (96.1%)	68.5%		
			NGM + PACEI50L	189	41 (98.4%)	48.7%	15.24	<0.0001
		2	NGM	113	43 (94.7%)	68.1%		
			NGM + PACEI50L	105	43 (99.1%)	21.9%	40.92	<0.0001
		3	NGM	102	41 (95.1%)	65.7%		
			NGM + PACEI50L	80	43 (97.5%)	56.2%	4.386	0.0362
PKH11	0.5	1	NGM	110	41 (98.2%)	70.0%		
			NGM + PKH11	127	43 (96.9%)	59.1%	3.845	0.0499
		2	NGM	113	43 (94.7%)	68.1%		
			NGM + PKH11	109	43 (97.2%)	55.1%	5.692	0.0170
	0.1	1	NGM	113	41 (98.2%)	68.8%		
			NGM + PKH11	110	43 (98.2%)	32.7%	7.643	0.0057
		2	NGM	113	43 (94.7%)	68.1%		
			NGM + PKH11	123	43 (99.2%)	30.9%	32.09	<0.0001
		3	NGM	106	41 (98.1%)	76.6%		
			NGM + PKH11	102	43 (95.1%)	64.7%	32.71	<0.0001
		4	NGM	104	41 (98.1%)	76.6%		
			NGM + PKH11	99	43 (98.0%)	61.6%	39.88	<0.0001
PACEI50L-v1	0.1	1	NGM	181	43 (96.1%)	68.5%		
			NGM + PACEI50L-v1	200	41 (98.0%)	62.5%	0.5108	0.4748
		2	NGM	102	41 (95.1%)	65.7%		
			NGM + PACEI50L-v1	121	41 (91.7%)	71.9%	1.7 × 10^−7^	0.9997
		3	NGM	113	41 (98.2%)	48.7%		
			NGM + PACEI50L-v1	134	41 (93.3%)	53.7%	0.5045	0.4775
PACEI50L-v2	0.1	1	NGM	181	43 (96.1%)	68.5%		
			NGM + PACEI50L-v2	195	41 (91.3%)	74.9%	7.489	0.0062
		2	NGM	102	41 (95.1%)	65.7%		
			NGM + PACEI50L-v2	108	41 (90.7%)	75.9%	0.0979	0.7543
		3	NGM	113	41 (98.2%)	68.8%		
			NGM + PACEI50L-v2	114	41 (94.7%)	68.4%	7.296	0.0069
PKH11-v1	0.1	1	NGM	106	41 (98.1%)	76.6%		
			NGM + PKH11-v1	100	43 (98.0%)	52.0%	42.14	<0.0001
		2	NGM	104	41 (98.1%)	76.6%		
			NGM + PKH11-v1	100	43 (98.9%)	64.0%	23.16	<0.0001

^a^ Analyses were performed with a paired log Rank survival test (survival curves are significantly different with respect to nematode growth medium (NGM) when *p* < 0.05). ^b^ Time (hours) of onset paralysis and % of non-paralyzed worms in brackets. ^c^ Percentage of paralyzed worms at 49 h.

**Table 5:** Effects of the PACEI50L, PACEI50L-v1, and PACEI50L-v2 treatments at 0.1 µg/mL on CL4176 paralysis and statistical analyses of the paralysis curves ^a^.

Treatment	Onset Paralysis ^b^	Paralyzed Worms ^c^	Log Rank X^2^	*p*-Value (vs. NGM)	*p*-Value (vs. PACEI50L)
NGM	41 (98%)	67.5			
NGM + PACEI50L	41 (99%)	42.3	9.419	0.0021	
NGM + PACEI50L-v1	41 (94%)	62.8	4.5 × 10^−5^	0.9947	0.0022
NGM + PACEI50L-v2	41 (92%)	73.1	2.245	0.1341	<0.0001

^a^ Analyses were performed with a paired log Rank survival test (survival curves are significantly different with respect to control NGM or PACEI50L when *p* < 0.05). ^b^ Time (hours) of onset paralysis and % of non-paralyzed worms in brackets. ^c^ Percentage of paralyzed worms at 49 h.

**Table 6:** Effects of the PKH11 and PKH11-v1 treatments at 0.1 µg/mL on CL4176 paralysis and statistical analyses of paralysis curves ^a^.

Treatment	Onset Paralysis ^b^	Paralyzed Worms ^c^	Log Rank X^2^	*p*-Value (vs. NGM)	*p*-Value (vs. PKH11)
NGM	41 (99%)	67.5			
NGM + PKH11	43 (98%)	47.5	19.26	<0.0001	
NGM + PKH11-v1	43 (99%)	58.0	9.665	0.0019	0.1808

^a^ Analyses were performed with a paired log Rank survival test (survival curves are significantly different with respect to control NGM or PKH11 when *p* < 0.05). ^b^ Time (hours) of onset paralysis and % of non-paralyzed worms in brackets. ^c^ Percentage of paralyzed worms at 49 h.

## Abbreviations

AD	Alzheimer’s disease
Aβ	Amyloid β peptide
Aβ_1–42_	Amyloid β peptide 1–42
ACE	Angiotensin converting enzyme
βAPP	β-amyloid precursor protein
BCA	Bicinchoninic acid
HPLC MS/MS	High-performance liquid chromatography tandem mass spectrometry
HSD	Honestly significant difference
LF	Lactoferrin
LFH	Lactoferrin hydrolysate
NGM	Nematode growth medium
PEP	Prolyl endopeptidase
POP	Prolyl oligopeptidase
SD	Standard deviation
